# Novel Risk Factors for Premature Peripheral Arterial Occlusive Disease in Non-Diabetic Patients: A Case-Control Study

**DOI:** 10.1371/journal.pone.0037882

**Published:** 2013-03-22

**Authors:** Annie M. Bérard, Aurélie Bedel, Rémi Le Trequesser, Geneviève Freyburger, Alan Nurden, Sylvie Colomer, Viviane Guérin, Marie-Christine Vergnes, François Becker, Gabriel Camelot, Luc Bressolette, Philippe Lacroix, Jean-Pierre Cambou, Alessandra Bura-Rivière, Joseph Emmerich, Michel Darmon, Anne-Marie Deletraz, Samir Mesli, Brigitte Colombies, Virginie Vanbrugghe, Claude Conri, Joël Constans

**Affiliations:** 1 Service de Biochimie, CHU Bordeaux, Bordeaux, France; 2 Equipe de Recherche Universitaire « Facteurs de risque vasculaires », Université Bordeaux, Bordeaux, France; 3 Usines FORD, Blanquefort, France; 4 Service d'Hématologie, CHU Bordeaux, Bordeaux, France; 5 CNRS-CHU Bordeaux, Bordeaux, France; 6 Service de Chirurgie Vasculaire, de Médecine Interne et de Chirurgie Générale, CHU de Besançon, Besançon, France; 7 Service de Médecine Interne et Pneumologie, CHU de Brest, Brest, France; 8 Service de Chirurgie Thoracique et Cardiovasculaire et Angiologique, CHU de Limoges, Limoges, France; 9 Service de Médecine Vasculaire, CHU de Toulouse, Toulouse, France; 10 Service de Médecine Vasculaire, Hôpital Européen Georges Pompidou, Paris, France; 11 Service de Médecine Vasculaire et Médecine Interne, CHU Bordeaux, Bordeaux, France; Goethe University, Germany

## Abstract

**Background:**

This study aimed to determine the prevalence of genetic and environmental vascular risk factors in non diabetic patients with premature peripheral arterial disease, either peripheral arterial occlusive disease or thromboangiitis obliterans, the two main entities of peripheral arterial disease, and to established whether some of them are specifically associated with one or another of the premature peripheral arterial disease subgroups.

**Methods and Results:**

This study included 113 non diabetic patients with premature peripheral arterial disease (diagnosis <45-year old) presenting either a peripheral arterial occlusive disease (N = 64) or a thromboangiitis obliterans (N = 49), and 241 controls matched for age and gender. Both patient groups demonstrated common traits including cigarette smoking, low physical activity, decreased levels of HDL-cholesterol, apolipoprotein A–I, pyridoxal 5′-phosphate (active form of B6 vitamin) and zinc. Premature peripheral arterial occlusive disease was characterized by the presence of a family history of peripheral arterial and carotid artery diseases (OR 2.3 and 5.8 respectively, 95% CI), high lipoprotein (a) levels above 300 mg/L (OR 2.3, 95% CI), the presence of the *factor V Leiden* (OR 5.1, 95% CI) and the *glycoprotein Ia^807T,837T,873A^* allele (OR 2.3, 95% CI). In thromboangiitis obliterans group, more patients were regular consumers of cannabis (OR 3.5, 95% CI) and higher levels in plasma copper has been shown (OR 6.5, 95% CI).

**Conclusions:**

According to our results from a non exhaustive list of study parameters, we might hypothesize for *1)* a genetic basis for premature peripheral arterial occlusive disease development and *2)* the prevalence of environmental factors in the development of thromboangiitis obliterans (tobacco and cannabis). Moreover, for the first time, we demonstrated that the *807T/837T/873A* allele of platelet glycoprotein Ia may confer an additional risk for development of peripheral atherosclerosis in premature peripheral arterial occlusive disease.

## Introduction

Among peripheral arterial diseases (PAD), lower limb peripheral arterial occlusive disease (PAOD) is considered as the disease process resulting from obstruction of large peripheral arteries, exclusive of the coronary and intracranial cerebrovascular system, most commonly due to atherosclerosis. PAOD which affects up to 20% of adults older than 50–55 years [1], is an important healthcare problem in Western countries and is associated with considerable morbidity and mortality [2]. The course of atherosclerosis is even more aggressive in young patients than in older ones with more frequent surgical interventions and death, and also more frequent coronary artery disease (CAD) [3], [4]. The prevalence of premature PAOD is estimated at 1% of the population. A number of vascular risk factors for premature PAOD have been demonstrated including cigarette smoking [5], high level of lipoprotein (a) (Lp(a)) [6], or fibrinogen [6] and other prevalent conditions such as chronic renal insufficiency [7], CAD [6], [8] or hypertension [6]. However, these risk factors fail to explain all the cases of PAOD in young adults, where the disease is thought to be multifactorial. The concerted action of both genetic and environmental factors has been incriminated and a family history of vascular disease is noted in most of the patients [9]. On the other hand, although PAOD concerns large peripheral arteries, thromboangiitis obliterans (TAO) affects small and medium sized peripheral blood vessels. Prevalence of TAO as a percentage of all cases of premature PAD (PPAD) is 0.5 to 5.6% in Western countries. Smoking is considered as the major risk factor [10], and a higher frequency of prothrombin gene mutation and endothelial dysfunction [11], [12], have also been reported in these patients. But TAO etiology remains mostly unelucidated.

The aim of the present study was to investigate genetic and environmental factors in two groups of young patients with either PAOD or TAO (diagnosis before 45-year old) and in one group of controls matched for age and gender. The prevalence of traditional and novel risk factors was compared in the three groups of subjects to define which of them are altered in PPAD when compared to controls and to recognize whether some of them are specifically associated with one or another of the PPAD subgroups.

## Methods

### Ethics Statement

The study protocol was approved by the Commitee to Protect Persons (CPP) from Bordeaux hospital in 1999 (Nb 99–35) and by the French Ministry of Health and Solidarity in 2001 (Nb DGS:2001–0058). Written informed consent was obtained from all study participants.

### Participants

A French, multicenter, age- and sex-matched case-control study was conducted between 2002 and 2007 including 113 patients with known PPAD (first observed before 45-year old) and 241 controls aged from 18 to 50 years. Since the definition of PPAD is controversial with respect to extracranial carotid circulation, upper extremity arteries, and mesenteric and renal circulation, we restricted our study on patients with chronic arterial occlusive disease in the arteries of the lower limbs. At the time of inclusion, all PPAD patients had symptoms of intermittent claudication (Fontaine stage II) or chronic critical limb ischemia (Fontaine stage IV) and an ankle-brachial index (ABI) at rest of less than 0.9, calculated according to AHA recommendations [13] or toe systolic pressure below 50 mm Hg or transcutaneous oxygen measurement below 30 mm Hg in case of uncompressible arteries. All patients had an angiographically confirmed diagnosis of PPAD. Patients with TAO were included according to Adar's criteria, *i.e*. possible diagnosis in the presence of sub-popliteal ischemia and tobacco consumption but in the absence of connective tissue disease, embolic disease, diabetes, dyslipidemia or hematologic disease; and likely diagnosis if recurrent venous thrombosis or Raynaud's phenomenon or upper limb ischemia. Unlike original Adar's criteria, we decided to include female patients as well. Workers in the Ford factory located in Blanquefort (33290– France) were enrolled as controls with no evidence of cardiovascular disease and an ABI above 1.0. All participants were asked to complete the same standardized questionnaire on alcohol consumption, use of cannabis (interview completed by 24 h-urinary detection of tetrahydrocannabinol (THC) by gas phase chromatography), medication use and a traditional/nontraditional cardiovascular risk factor evaluation (family and personal history of cardiovascular diseases (CVD), hypertension, cigarette smoking, body mass index (BMI), exercise/physical activity). The family history was collected from each proband by personal interview about the occurrence of CAD, cerebrovascular disease and PPAD in each of their first-degree relatives before the age of 55 years for men and of 65 years for women. All included patients underwent clinical and instrumental evaluation comprising electrocardiogram, treadmill exercise, duplex ultrasound of cervical arteries and abdominal aorta. The study subjects were considered to be hypertensive if systolic arterial pressure exceeded 140 mm Hg and/or diastolic arterial pressure exceeded 90 mm Hg [14] or if they used antihypertensive drugs. Information on cigarette smoking status (current, former, never) and amount (pack x years) was estimated from interview and completed by urinary cotinin determination using high-performance liquid chromatography. BMI (kg/m^2^) was calculated. Physical activity was estimated as never/sometimes/intense. The term of “sometimes” was attributed to adults who did at least 150 minutes of moderate-intensity aerobic physical activity throughout the week, or do at least 75 minutes of vigorous-intensity aerobic physical activity throughout the week, or an equivalent combination of moderate- and vigorous-intensity activity; the term of “intense” was used for people having exercise or playing a sport daily.

We excluded participants with diabetes as a major risk of PAD (based on glycemia and glycated hemoglobin results), active infection, chronic inflammatory disease, auto-immune pathology, thyroid abnormalities, renal and/or liver failure, history or presence of cancer, pregnant women, and those unable or unwilling to sign the written informed consent that was systematically required.

### Blood Analysis

Blood samples were taken from all study participants under standardized conditions after an overnight fasting period. Serum and plasma samples were collected and frozen at −80°C before analysis except for lipid determination. Parameters were measured in the hospital laboratories of Bordeaux (France) as previously described [15]: ***1*** **/**fasting glucose as well as creatinine, ALAT/ASAT/LDH/GGT and TSH for renal, liver and thyroid functions respectively; ***2*** **/**total cholesterol, direct HDL-cholesterol and triglycerides, the LDL-cholesterol level being either calculated by the Friedewald equation when triglycerides were <306 mg/dL or determined by direct method, apolipoproteins (apo) A–I and B, Lp(a); ***3*** **/**inflammatory markers: high-sensitivity CRP (hsCRP) and fibrinogen (using the Fibriquick® reagent bioMerieux according to Clauss technique); ***4*** **/**determination of homocystein based on microparticle enzyme immunoassay technology (Roche, Meylan, France) as well as pyridoxal 5′ -phosphate (active form of B6 vitamin) by high-performance liquid chromatography, B9 and B12 vitamins by competitive chemiluminescent immunoassay (Roche, Meylan, France); ***5*** **/**zinc, copper and selenium in blood samples evaluated by flame atomic absorption spectrophotometric method; ***6*** **/**hemostatic markers: fibrinogen, antithrombin, proteins C and S by routine methods (Coamatic® protein C, Chromogenix, Mölndal, Sweden, IL test® protein S kit and IL test® antithrombin, Instrument Laboratory Company, Lexington, MA), and antiphospholipids as previously described [16].

### DNA Analysis

Genomic DNA was isolated from buffy coat prepared from EDTA whole-blood samples by using the commercially available Qiagen DNA isolation kit (Courtaboeuf, France) and was kept at +4°C until further assessment. We selected candidate genes encoding proteins that are potentially contributing to the atherosclerotic process as known in premature CAD. We particularly focus on the biological aspects of variants and mutations of these genes which impact on: ***1)*** lipid and lipoprotein metabolism via the intracellular incorporation of lipids in lipoproteins during synthesis (microsomial triglyceride transfer protein: *MTTP^−493G/T^*(rs1800591)), the remodelling of plasma lipoproteins through the plasma transfer of cholesterol from HDL to triglyceride-rich lipoproteins (cholesterol ester transfer protein: *CETP-T*aq*I* (rs708272)), specific membrane transporter systems as ABCA1 which allows the transfer of intracellular cholesterol to plasma HDL (*ABCA1^R219K^* (rs2230806)), molecule-mediated hepatic uptake of atherogenic lipoproteins as apoE (*APOE ε2/ε3/ε4* (*ε2* allele*^R158C^*, rs7412; *ε4* allele*^C112R^*, rs429358)), or transcription factors involved in lipid metabolism and atherogenesis as peroxisome proliferator-activated receptor γ (*PPARG^161C/T^* (rs3856806)); ***2)*** cell adhesion to vascular endothelium via E selectin (*SELE^S128R^* (rs5361)); and ***3)*** thrombosis via coagulation factors as factors II and V (*F2^20210G/A^* (rs1799963), *F5^R506Q^ Leiden* (rs6025)) as well as platelet glycoproteins Ia and IIIa (*ITGA2^807C/T, 837C/T, 873 G/A^* (rs1126643, rs 1139484 and rs1062535) and *ITGB3^L33P^* (*HPA-1*, rs5918)).

**Figure 1:**
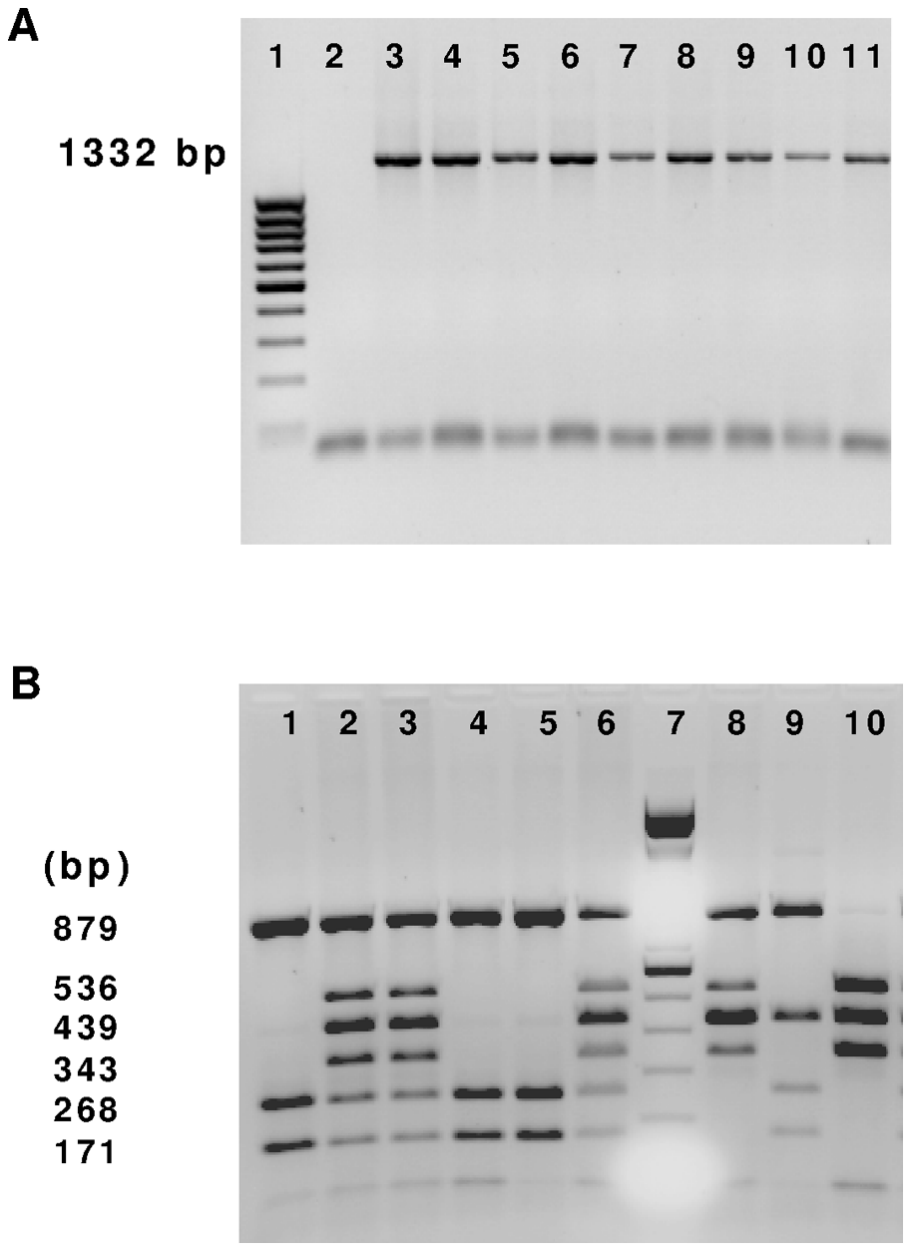
PCR analysis of genomic DNA encoding the glycoprotein Ia gene surrounding the 807, 837 and 873 polymorphisms. *(A)* Amplified products (1332 bp) were resolved by 1% agarose gel electrophoresis and stained with ethidium bromide. Lane 1: molecular weight marker; lane 2: blank; lanes 3 to 11: different genotyped individuals. *(B)* Analysis of *ITGA2^807C/T, 837C/T, 873 G/A^* polymorphisms by PCR-RFLP using *Bgl II* and *Asn I* endonucleases on 1.5% agarose gel. Lane 7: molecular weight marker; other lanes: different genotypes according to reference 29 (lanes 1, 4, 5: 2/2, lanes 2, 3, 6: 1/2, lane 7: 1/3, lane 8: 2/3, lane 9: 1/1).

Except for *MTTP*, *F2* and *F5* gene polymorphisms, DNA fragments containing the mutational sites were amplified by polymerase chain reaction (PCR) and digested with allele-specific restriction enzymes (specific primers and restriction enzymes are shown in Table S1 in Supplementary data). Fragments were separated on polyacrylamide (*APOE ε2/ε3/ε4*) or agarose gels according to their size, and visualized by use of ethidium bromide. An example of observed alleles is illustrated in Figure 1. As quality control of the method, 5 randomly chosen subjects were additionally genotyped by sequencing of PCR products using an ABI sequencer (Applied BioSystems, Courtaboeuf, France). For *MTTP* gene polymorphism (*MTTP^−493G/T^*, rs 1800591), amplified selected DNA fragments were sequenced. No new gene variation has been found by sequencing in our study. For the *F5^G1691A^ Leiden* (rs6025) and *F2^G20210A^* (rs1799963) polymorphism determination, the TaqMan® fluorogenic 5′→3′ nuclease assay (PE/Applied Biosystems, Foster City, CA) was carried out using the primers and probes of the human pre-designed TaqMan® SNP Genotyping Assays. In each run, blanks and known homozygotes and heterozygotes were included.

### Statistical Analysis

We compared risk factors distribution ***1)*** in controls versus PPAD patients, ***2)*** in PAOD versus TAO, ***3)*** in controls versus PAOD and ***4)*** in controls versus TAO. For comparing the values of continuous quantitative variables between groups of subjects, *t* tests were used. For comparing the percentages of qualitative categorical variables between groups of subjects, χ^2^ tests were used. Quantitative variables (expressed in tables as mean ± SD) had a normal distribution except in the case of cotinin and cigarette consumption (expressed as pack x years) since zero values were found in non-smokers. Qualitative variables are expressed in tables in percentages. Hardy-Weinberg equilibrium of allele frequencies was tested for each SNP in controls by χ^2^ statistics. A Pearson χ^2^ test was performed to detect differences in SNP allele frequency distribution between patients and controls.

#### General Linear Models

Disease state (no disease, PPAD, PAOD, TAO), allele frequencies, and some other qualitative variables were defined as independent (explicative) variables. The effects of all categorical variables, including allele frequencies, on the dependent quantitative variables were tested systematically for the whole subject population using univariate general linear models. Age and sex were included as an adjustment factor. Before testing the effect of independent categorical variables on the dependent variables, interfering covariables (to be used as adjustment factors in the linear models) were identified by 2 types of tests: ***1)*** Quantitative variables chosen as adjustment factors: linear correlations were performed between all possible pairs of quantitative variables in the whole subject population on one hand, and in either controls, patients, PAOD or TAO separately on the other hand. All independent variables showing correlations significant either at the 0.01 level or at the 0.05 level with a clinical or biological significance, were retained as quantitative adjustment factors to be included in the linear models. This procedure was chosen to avoid the presence of too many covariables in the general linear models. ***2)*** Qualitative variables chosen as adjustment factors: all independent qualitative variables showing correlations at the 0.05 were retained as qualitative adjustment factors specifically for each studied dependent variable. Results of the General Linear Models include adjusted *p* values, means, and SD. For each of the biallelic polymorphisms, 100% of patients and controls were successfully genotyped. The allelic model was chosen for testing association between polymorphisms and the diseased or control phenotype. Alleles were in Hardy-Weinberg equilibrium and the lower degree of freedom (*df*) required made it more powerful than the general genetic model. The general model, or allele-based test, requiring 2 *df*, was used. SNP were then analyzed by comparing differences in allele frequencies between cases and controls using a χ^2^ test. Statistical analyses were performed using the SPSS statistical package (SPSS Inc., Chicago, IL, USA).

Confidence intervals (CI) for proportions were calculated using the Wilson score method without continuity correction [17]. CI for the odds ratios (OR) were calculated using the methods described by Armitage and Berry [18].

## Results

### Population Characteristics

Population characteristics are summarized in Table 1. *P* values from comparison between controls and PPAD and between PAOD and TAO are directly given in Table 1. *P* values from comparison between PAOD or TAO and controls are given in the text.

**Table 1:** Clinical Characteristics of the Study Sample.

Study characteristics	Controls	PPAD	p-value	PAOD	TAO	p-value
	n = 241	n = 113	PPAD vs controls	n = 64	n = 49	PAOD vs TAO
**Age at the inclusion time (years)**	33.1±6	39±7.8	<0.001	41±6.9	36.4±8.2	0.002
**Sex (% men)**	77.2	78.8	ns	78.1	79.6	ns
**Fontaine's stage (diagnostic time)**						<0.001
– II (%)	-	54.8		79.7	22.5	
– III (%)	-	19.5		14	26.5	
– IV (%)	-	25.7		6.3	51	
**Fontaine's stage (inclusion time)**						<0.001
– II (%)	-	46		71.9	12.2	
– III (%)	-	17.7		14.1	22.4	
– IV (%)	-	36.3		14	65.4	
**Associated arterial diseases (%)**			<0.001			<0.001
– 0	98.7	70		53.2	91.8	
– 1	1.3	26.5		40.6	8.2	
– 2 or more	0	3.5		6.2	0	
– Occlusive carotid artery disease	0	25.9	<0.001	40.6	6.2	<0.001
– Coronary disease	1.2	8.8	<0.001	14.1	2	0.03
– Aortic abdominal anevrysm	0	0.9	ns	1.6	0	ns
**Other associated vascular diseases (%)**						
– Raynaud's disease	1.7	24.7	<0.001	6.3	49	<0.001
– Venous thromboembolism	2.5	19.5	<0.001	12.5	28.6	0.03
– None	95.9	71.7	<0.001	81.2	38.8	<0.001
**Vascular surgery history (%)**	2.5	53.1	<0.001	62.5	40.8	ns
**Medication use (%)**						
– Statins	2	30	<0.001	40.6	8.2	<0.001
– Anti-platelet agents:						
– Aspirin	0	48.7	<0.001	42.1	57.1	ns
– Clopidogrel	0	26.5	<0.001	39.1	10.2	0.001
– Aspirin + Clopidogrel	0.4	3.5	ns	6.25	0	ns
– Iloprost	0	9.7	<0.001	1.6	20.4	0.001
– Statins + antiplatelet agents	0.4	23.9	<0.001	37.5	6.1	<0.001
– none	97.5	16.8	<0.001	12.5	22.4	ns

Results as mean ± SD; ns: no significant different when p>0.05.

(PPAD, premature peripheral arterial diseases; PAOD, peripheral arterial occlusive disease; TAO, thromboangiitis obliterans).

Age at the inclusion time was higher in PPAD patients than in controls, with a significant difference between PAOD patients and controls. Age was thus included in the adjustment for statistical analysis. At diagnosis time, a 5-year difference was present between PAOD and TAO patients showing the earlier diagnosis of the latter (38.3±5.7 vs 33.4±7.6 years, p<0.03). Women represented ≈21% in all groups of subjects with no difference between the groups. This result is new compared to other studies, probably because those scrupulously considered Adar's criteria.

According to Fontaine's stages, most of PPAD had intermittent claudication (stage II) at the time of inclusion as well as at the time of diagnosis. But the distribution of the stages was significantly different between patients with TAO with a large predominance of rest ischemia (stage IV), and PAOD cases mainly at a claudication stage (stage II). This distribution between the two groups of patients was the same at the inclusion and diagnosis times with more cases at stage IV (rest ischemia) in both study groups at the inclusion time. Moreover, 30% of the 113 patients had other arterial diseases (occlusive carotid artery disease, coronary disease) compared to controls (1.3%). However, the prevalence of arterial diseases, mainly carotid and coronary artery diseases, was higher in patients with PAOD than in those with TAO (46.8 vs 8.2%). On the other hand, the prevalence of Raynaud's disease and venous thromboembolism was significantly higher in PPAD patients vs controls (p<0.001) but a marked predominance was found in patients with TAO (61.2 vs 18.8%, p<0.001). Because of their vascular disease, most of PAOD and patients with TAO underwent a vascular surgery or amputation and used medication (p<0.001). For this last point, according to their pathology and other diseased vascular districts, statins and clopidogrel were mainly used by PAOD patients whereas iloprost was predominantly given to patients with TAO. No difference was demonstrated for aspirin intake between the two patient groups.

### Cardiovascular Risk Factor Analysis

Table 2 shows the distribution of vascular risk factors among individuals with or without PPAD as well as among PAOD and patients with TAO. *P* values from comparison between controls and PPAD and between PAOD and TAO are directly given in Table 2. *P* values from comparison between PAOD or TAO and controls are given in the text.

**Table 2:** Cardiovascular Risk Factors in the Study Sample.

Study characteristics	Controls		PPAD	p-value	PAOD	TAO		p-value
	n = 241		n = 113	PPAD vs controls	n = 64	n = 49		PAOD vs TAO
**Family history (1^st^-degree relatives)**								
**Arterial diseases (%):**								
– Peripheral arterial disease	10		17.8	0.04	21.3	13		ns
– Occlusive carotid artery disease	2.1		7.5	0.015	11.4	2.17		ns
– Coronary disease	28.2		26.2	ns	36.1	13		0.007
– Aortic abdominal anevrysm	1.3		1.9	ns	0	4.4		ns
– None	68.9		63.7	ns	56.3	73.5		ns
**Others vascular diseases (%):**								
– Raynaud's disease	2.1		2.8	ns	1.7	4.3		ns
– Venous thromboembolism	15		16.8	ns	16.4	17.4		ns
– None	83		82.3	ns	82.8	81.6		ns
**Patient characteristics**								
**Physical exercice (%):**				<0.001				ns
– Never	24.9		52		47.6	58.6		
– Sometimes	57.7		27		33.3	17.2		
– Intense	17.4		21		19.1	24.2		
**Body mass index (kg/m^2^)**	24.5±3.5		24.5±3.6	ns	24.6±3.8	24.4±3.5		ns
**Hypertension (%)**	48.5		32.1	0.004	42.6	18.8		0.008
**Smoking status (%):**				<0.001				ns
– Never	31.1		6.2		4.7	8.2		
– Former	34		27.4		32.8	20.4		
– Current	34.9		66.4		62.5	71.4		
**Pack years**	6.7±7.4		24.6±16.6	<0.001	26.4±17.7	22.4±15		ns
**Urinary cotinine (µg/dL)**	58±99		103±127	0.013	113±122	94±136		ns
**Urinary THC** * **(%)**	5.5		9	ns	2.5	17.1		0.03
**Biology:**								
Triglycerides (mg/dL)	140±96		149±114	ns	166±140	140±52		ns
Total cholesterol (mg/dL)	209±39		197±46	0.013	197±50	201±39		ns
HDL-cholesterol (mg/dL)	54.2±11.6		46.4±11.6	<0.001	42.5±11.6	46.4±11.6		ns
LDL-cholesterol (mg/dL)	131.6±31		124±38.7	ns	120±38.7	127.7±35		ns
Total chol/HDL-chol	4.1±1		4.6±1.5	ns	4.7±1.6	4.6±1.2		ns
ApoA-I (mg/dL)	150±20		130±30	0.001	130±30	130±30		ns
ApoB (mg/dL)	90±20		90±30	ns	90±30	100±30		ns
ApoA-1/ApoB	1.7±0.5		1.5±0.6	ns	1.5±0.6	1.5±0.5		ns
Lp(a)† (mg/dL)	25.7±25.4		46.8±25.4	ns	57.5±63.4	32.5±32.3		0.02
hsCRP‡ (mg/L)	2.3±4.8		6.6±9.2	0.015	4.7±6	9.3±12		ns
Fibrinogen (mg/dL)	330±60		360±10	ns	340±90	380±120		ns
Homocystein (µmol/L)	12.8±5.8		12±5.6	ns	12.1±6.1	11.4±4.9		ns
B6 vitamin (active form) (nmol/L)	50.6±20.4		27.5±15.8	<0.001	28.1±15.1	25.1±15.9		ns
B9 vitamin (nmol/L)	12.2±4.8	12.3±7.1		ns	12.4±8.2		12.1±5.8	ns
B12 vitamin (pmol/L)	337±123	387±217		0.016	381±178		393±257	ns
Plasma Copper (µmol/L)	16.1±4.5		17.8±4.5	0.001	16.6±3.4	19.2±5.2		0.03
Plasma Selenium (µmol/L)	1.1±0.2		0.9±0.2	ns	1.0±0.2	0.9±0.2		ns
Plasma Zinc (µmol/L)	14.6±2.4		13.2±2.7	0.003	13.3±2.7	13.2±2.7		ns

**Results as mean ± SD; ns: no significant different when p>0.05.**

* THC: tetrahydrocannabinol, † Lp(a): lipoprotein(a), ‡ hsCRP: ultrasensible C-reactive protein.

(PPAD, premature peripheral arterial diseases; PAOD, peripheral arterial occlusive disease; TAO, thromboangiitis obliterans).

The prevalence of early cardiovascular events occurring in first-degree relatives of subjects with PPAD was determined. Interestingly, peripheral and carotid arteries were more often altered in first-degree relatives of PPAD patients than in controls, while coronary arteries were not. Moreover, when first-degree relatives of PAOD and patients with TAO were separately compared to controls, the former had more peripheral and carotid artery diseases (p<0.02 and p<0.002 respectively), while the latter had fewer coronary diseases (p<0.035). Finally, the prevalence of venous thromboembolism in first-degree relatives was not significantly different among study groups.

Among all other evaluated vascular risk factors, hypertension and hyperhomocysteinemia were not more prevalent in both groups of patients compared to controls. Lack of physical exercise, cigarette smoking, low plasma HDL-cholesterol and apoA-I, and high sensitive CRP (hsCRP) were found in PPAD more frequently than in controls. Absence of physical activity was significantly more frequent in both patient groups compared to controls (p<0.006 and p<0.001 respectively), but no significant difference was detected for BMI. Active cigarette smoking was far more prevalent in both PAOD and TAO groups than in controls as assessed by claimed consumption (p<0.001 for both groups) or urinary cotinin evaluation (p<0.004 and p<0.05 respectively). In addition, cannabis consumption was higher in TAO group than in PAOD patients and controls as assessed by interview and positive urinary tetrahydrocannabinol (THC) detection (p<0.015). On the other hand, HDL-cholesterol and apoA-I were significantly lower in both groups of patients compared to controls (p<0.001 for both variables in PAOD patients, p<0.004 and p<0.05 respectively in patients with TAO) while Lp(a) showed higher levels in PAOD patients only (p<0.02). It is noteworthy that there was no difference in LDL-cholesterol probably due to statin medication. Two inflammatory factors, hsCRP and fibrinogen, were also evaluated and showed higher plasma concentrations in patients with TAO only, when compared to controls (p<0.02 and p<0.03 respectively); this is presumably due to local infection found in many patients with toe necrosis. Finally, homocystein metabolism evaluation demonstrated a decrease in pyridoxal 5′-phosphate (active form of B6 vitamin) in both groups of patients compared to controls (p<0.005). On the other hand, as copper, selenium and zinc are essential cofactors for antioxidative enzymes, i.e. Cu-Zn superoxide dismutase and glutathione peroxidase, we investigated the relationship between these trace-elements and PPAD. We showed an increase in plasma copper levels in patients with TAO (p<0.001) and a decrease in plasma zinc levels in PAOD (p<0.02) and TAO (p<0.004) patients compared to controls so that both groups of cases had diminished zinc/copper ratio (p<0.001). Nevertheless, concentrations in copper and zinc remained in the normal range. At last, no difference in the frequency of antiphospholipid antibodies was shown among the groups (data not shown).

### Polymorphism Frequency

The distribution of all genotypes in the different study groups is depicted in Table 3 and was in Hardy-Weinberg equilibrium. When comparing differences in allele frequencies between cases and controls, only *F5^R506Q^ Leiden* (rs6025) and *ITGA2^807C/T, 837C/T, 873 G/A^* (rs1126643, rs 1139484, rs1062535) were significantly different in PPAD patients compared to controls. Interestingly, a statistically significant different distribution was found between PAOD and controls with an overrepresentation of *F5 Leiden*, *ITGA2^807T,837T,873A^* (allele 1) and *PlA2* allele of *ITGB3^L33P^* (rs5918) in PAOD patients, and more TAO patients heterozygous for the *F2^20210G/A^* (rs1799963) were found compared to controls.

**Table 3:** Gene Polymorphisms in the All Study Population.

	Controls	PPAD	p-value	PAOD	p-value	TAO	p-value
	%	%	PPAD vs controls	%	PAOD vs controls	%	TAO vs controls
***MTTP^−493G/T^ (rs1800591)***G alleleT allele	27.6 72.4	29.0 71.0	ns	31 69	ns	27.2 72.8	ns
***CETP-TaqI (rs708272)***B1 alleleB2 allele	43.5 56.5	36.5 63.5	ns	34.5 65.5	ns	39.2 60.8	ns
***ABC-A1^10521G/A^*** ***(rs2230806, intron 8)***G alleleA allele	71.9 28.1	72.9 27.1	ns	74.2 25.8	ns	71.8 28.2	ns
***APOE***ε2 *(rs7412)*ε3ε4 *(rs429358)*	6.3 82.111.6	5.6 83.6 10.8	ns	4.3 84.5 11.2	ns	6.5 83.7 9.8	ns
***PPARG^161C/T^(rs3876806)***T alleleC allele	88.6 11.4	88.3 11.7	ns	89.7 10.3	ns	87.0 13.0	ns
***SELE^561A/C^ (rs5361)***A alleleC allele	88.0 12.0	85.5 14.5	ns	82.8 17.2	ns	89.1 10.9	ns
***F2^20210G/A^ (rs1799963)***G alleleA allele	98.4 1.7	96.8 3.3	ns	97.4 2.6	ns	95.8 4.2	0.03
***F5^1691G/A^ Leiden (rs6025)***G alleleA allele	99.0 1.0	95.5 4.5	0.003	95.0 5.0	0.003	97.9 2.1	ns
***ITGA2 (gpIa^807C/T, 837C/T, 873 G/A^*** * ***^)^*** ***(rs1126643, 1139484, 1062535)***allele 1allele 2allele 3	35.7 50.214.1	47.6 44.4 8.0	0.001 ns 0.02	50.0 44.8 5.2	0.001 ns 0.01	43.5 45.6 10.9	ns ns ns
***ITGB3^L33P^ (gpIIIa)*** ***(rs5918)***Pl^A1^Pl^A2^	80.4 19.6	81.4 18.6	ns	78.4 21.6	0.01	83.7 16.3	ns

* allele 1 = 807T/837T/873A, allele 2 = 807C/837T/873G, allele 3 = 807C/837C/873G

ns: no significant different when p>0.05.

(PPAD, premature peripheral arterial diseases; PAOD, peripheral arterial occlusive disease; TAO, thromboangiitis obliterans).

## Discussion

This case-control study clearly demonstrated common and particular PAD risk factors (Table 4), at least among those determined in this study, for patients presenting either a PAOD or a TAO, after adjustment for potential confounders.

**Table 4:** Risk Factors for PAOD Patients and TAO.

Parameters	PAOD (n = 64) vs controls (n = 241)	TAO (n = 49) vs controls (n = 241)
	OR [95% CI]	OR [95% CI]
**Physical exercice** (never)	**2.7**† [1.4–5.4]	**4.3** * [1.9–9.5]
**Smoking** (current or former)	**9.2** * [2.8–30.2]	**5.1** * [1.8–14.6]
**Package x year number **>17 >13	**17.1** * [8.7–33.6]	**15.9** * [7.4–34.3]
**Urinary cotinin** >50 ng/mL	**3.3** * [1.7–6.3]	**3.6** * [1.7–7.7]
**HDL-cholesterol** <40 mg/dL	**5.2** * [2.7–10.1]	**5.2** * [2.5–10.6]
**ApoA-I** <130mg/dL	**4.9** * [2.7–8.9]	**4.3** * [2.2–8.1]
**ApoA-I/ApoB** <1	**5.7** * [2.15–15.2]	**6.7** * [2.5–18.5]
**B6 vitamin (active form)** <20 nmol/L	**23.4** * [5.14–107]	**38.3** * [8.1–18]
**Plasma zinc** <12 µmol/L	**3.4** * [1.63–7]	**3.9** * [2–7.4]
**Plasma zinc/copper ratio** <0.7	**2.6**† [1.4–5.1]	**5.1**† [2.6–10]
**Family history** Peripheral arterial disease Occlusive carotid artery disease	**2.3**‡ [1.1–4.8] **5.8**† [1.8–18.9]	ns ns
**Lp(a)** >30 mg/dL	**2.3** * [1.3–4]	ns
**Factor V Leiden** presence	**5.1**† [1.5–17.4]	ns
**gpIa allele 1** presence	**3.0**† [1.5–5.9]	ns
**gpIa allele 3** presence	**0.3**† [0.13–0.8]	ns
**Urinary THC** >50 ng/mL	ns	**3.5**‡ [1.3–10]
**hsCRP** >0.24 mg/dL	ns	**8.2** * [4.1–16.3]
**Fibrinogen** >370 mg/dL	ns	**3.4** * [1.7–7.7]
**Plasma copper** >18 µmol/L	ns	**6.5** * [3.3–12.7]

* p<0.001, †p<0.01, ‡ p<0.05, ns: no significant different when p>0.05.

(PAOD, peripheral arterial occlusive disease; TAO, thromboangiitis obliterans).

Both patient groups demonstrated common clinical conditions associated with PPAD including low physical activity, cigarette smoking, decreased HDL-cholesterol and apoA-I levels, lower pyridoxal 5′-phosphate (active form of B6 vitamin) and zinc concentrations. Both patients with PAOD and TAO had reduced physical activity compared with controls but both populations of patients had a high frequency of claudication, rest pain, and lower extremity ulcers which represents a bias to conclude about this recognized risk factor. Cigarette smoking is widely accepted as a major risk for PAD [5] even in young patients [5], [9]. The underlying mechanisms explaining the tobacco-induced risk include alterations in HDL-cholesterol [19] and pyridoxal 5′-phosphate [20] levels, hemostatic factors [21] and endothelial function [22], all of them known as playing an important role in cardiovascular diseases. In both PPAD groups, we demonstrated a significant decrease in HDL-cholesterol, apoA-I and pyridoxal 5′-phosphate levels associated with a prothrombotic status. Indeed HDL lipoproteins play a pivotal role in the reverse cholesterol transport, known to have athero-protective effects. Nevertheless, their role is unknown in TAO. In addition, there was a significant inverse association between pyridoxal 5′-phosphate and hsCRP (data not shown, p<0.03), independently of homocystein level so far showing no significant difference. This relationship was also demonstrated elsewhere for ischemic stroke and coronary artery disease [23], [24]. Finally, patients with PPAD had significant lower zinc/copper ratio. This imbalance in zinc/copper metabolism may be a consequence of an acute phase response known in these patients and might contribute to the pathological process as mentioned elsewhere [25].

In PAOD patients, specific PAD risk factors such as higher Lp(a) levels, presence of *F5 Leiden* and *ITGA2* ^807T,837T,873A^ allele as well as a family history of peripheral and carotid artery diseases were demonstrated. As Lp(a) level is mostly genetically determined and is unaffected by statins, we were able to confirm the correlation between high Lp(a) and premature PAD in PAOD patients as described by others [26]. The pathophysiological role of Lp(a) is not well understood, but at least two properties may be attributed to Lp(a): *1)* once oxidized, Lp(a) contributes to foam cell formation; *2)* Lp(a) attenuates fibrinolysis and promotes coagulation [27]. Thrombophilic conditions could be also promoted by the presence of *F5 Leiden*, *F2^20210A^*, *ITGA2^807T,837T,873A^* and *ITGB3^PlA2^* alleles. In our study, premature PAD in PAOD patients was associated with an increased prevalence of *F5 Leiden* and *ITGA2^807T,837T,873A^* but not of *F2^20210A^* or *ITGB3^PlA2^* alleles. A recent meta-analysis on genetic association studies in PAD including 2,466 patients, selected independently to their age-of-onset and to the presence or not of diabetes, and 2,686 controls demonstrated that neither *F5* nor *F2 Leiden* mutations were risk factors for PAD [28]. In the present study, inclusion of non-diabetic patients with a mean age-of-onset under 45 years may explain the difference. The increased frequency observed for *F5* heterozygous mutation indicates that, in young patients, these common thrombophilia factors participate in uncovering underlying vessel alterations. In the older patients included in the meta-analysis, the prevalence (and probably the severity) of vessel alteration exceeds largely that of thrombophilia whose additive contribution in symptoms onset is no more detectable. *F5 Leiden* is a common gain-of-function mutation, which leads to resistance to activated protein C: it modifies one of the cleavage sites in factor V, where activated protein C inactivates factor Va. The delay in factor Va destruction facilitates overproduction of thrombin leading to excess fibrin generation and excess clotting. On the other hand, we demonstrated for the first time that polymorphisms in platelet glycoprotein Ia (*ITGA2^807T,837T,873A^*) are associated with peripheral arterial disease in premature PAOD. The *ITGA2^807T,837T,873A^* allele has been associated with increased levels of glycoprotein Ia-IIa (α2β1 integrin) on platelets which could enhance platelet adhesion to collagen and thus contribute to an increased risk of thrombosis. Indeed, patients with atherothrombotic manifestations have been demonstrated to have a higher frequency of *ITGA2^807T^* associated with higher levels of platelet glycoprotein Ia-IIa [29]. In addition, higher platelet activation has been shown in patients with lower limb ischemia [30] and the highest activation rate was found in patients with the most severe PAD [31]. Involving platelet pathophysiology in PAD is in line with the recognition of the importance of platelets in chronic inflammatory processes such as atherosclerosis. Altogether, our findings could explain the use of clopidogrel in PAOD patients. On contrary, the use of aspirin is more controversial but it may be explained more by its pleiotropic effects, including protection of LDL and fibrinogen from oxidation [32]. Taken together, all determined risk factors for premature PAD in PAOD patients (cigarette smoking, lack of physical activity, decreased HDL-cholesterol and apoA-I, high Lp(a) levels, hypercoagulable states) cannot explain the development of premature PAOD. Several lines of evidence indicate that susceptibility to premature atherosclerosis of the coronary and cerebral vasculature is heritable. In our study, premature symptomatic vascular disease (peripheral and occlusive carotid artery diseases) was significantly more prevalent in family members of premature PAOD patients than in family members of healthy individuals from the control group. Our results provide strong evidence for familial aggregation of arterial diseases in the first-degree relatives of these patients as described elsewhere [9]. It may therefore reflect the clustering in some families of genes (such as genes encoding hemostatic factors, Lp(a) or platelet glycoproteins) that confer susceptibility to atherosclerosis. Thus premature PAOD patients seem to be genetically susceptible to the effects of environmental factors such as cigarette smoking and sedentary lifestyle.

TAO patients were shown to present specific vascular risk factors such as cannabis consumption, higher copper concentration as well as higher hsCRP and fibrinogen levels. This last point, i.e. inflammation, has already been discussed above with a possible bias due to their pathology and associated with diminished functional capacity. In respect of cannabis consumption, cannabis arterial disease has been described [33] and a vasoconstrictor effect of THC seems probable. Furthermore cannabis consumption is often associated to tobacco smoking [34] and a synergistic deleterious effect of cannabis and tobacco seems likely, along with that of a probable common contaminant such as arsenic. As a matter of fact, a positive relationship between plasmatic arsenic levels and the severity of TAO has been demonstrated [35]. In addition, *Cannabis sativa* has a very high capability to absorb and accumulate heavy metals such as copper [36]. In the present study, patients with TAO presented higher plasma copper concentrations. Thus, environmental factors such as cannabis consumption associated with cigarette smoking may play a major role in the development and progression of the vascular disease in patients with TAO.

In conclusion, our present study demonstrated that the risk of developing premature PAD was significantly increased in current or former smokers versus non-smokers, in cases with low plasma concentrations in HDL-cholesterol (below 0.40 mg/dL) and apoA-I (below 130 mg/dL), active form of B6 vitamin (below 20 nmol/L) and zinc (below 12 µmol/L with a zinc/copper ratio less than 0.7). Moreover each group of patients showed specific risk factors. In one hand, our results support the importance of environmental factors in the development of TAO. Particularly our case-control study shows more frequent cannabis consumption in patients with TAO compared with controls or patients with premature PAOD. This result is particularly relevant even if more scientific reports need to be forthcoming to support the hypothesis of cannabis being a causative factor or co-factor of TAO. For this group of patients, it is also noticeable that female patients with PPAD may have clinical characteristics of TAO. It might be proposed that Adar's criteria for inclusion of patients with TAO should take the inclusion of female patients into account. On the other hand, high Lp(a) levels above 300 mg/L as well as the presence of a family history of peripheral and carotid artery diseases, the *F5 Leiden* and the *ITGA2^807T,837T,873A^* allele were specifically linked to premature PAOD. These results led us to hypothesize for a genetic basis for premature PAOD development. Moreover and for the first time, our study demonstrated differences in allelic frequencies of platelet glycoprotein Ia which seem to be associated with a different predisposition to atherothrombosis in premature PAOD.

## Supporting Information

Table S1 **List of Used Specific Primers and Restriction Enzymes.**
(DOC)
