# Supplementary material for: Novel Risk Factors for Premature Peripheral Arterial Occlusive Disease in Non-Diabetic Patients: A Case-Control Study
Source: PLoS One. 2013 Mar 22;8(3):e37882. doi: 10.1371/journal.pone.0037882 (PMC3606168; doi:10.1371/journal.pone.0037882)
Supplement: Table S1 — List of Used Specific Primers and Restriction Enzymes. (DOC) [file pone.0037882.s001.doc]

**Supporting information**

**TABLE S1. List of Used Specific Primers and Restriction Enzymes.**

| **Targeted genes** | **Specific primers and Tm**  Forward (F), Reverse (R) | **Restriction enzymes** |
| --- | --- | --- |
| ***MTTP^-493G/T^*** ***(rs1800591)*** | F : 5’-AGTTTCACACATAAGGCAAATCATCTA-3’ Tm=48,6°C  R : 5’-GTGGGAGGGTAGTAAGGATTCT-3’ Tm=52,2°C |  |
| ***CETP-TaqI (rs708272)*** | F : 5’-acatattaagcaattatccagat-3’ Tm=58°C  R : 5’-cacttgtgcaacccatacttgact-3’ Tm=68°C | ***TaqI*** |
| ***ABC-A1^10521G/A^***  ***(rs2230806,***  ***intron 8)*** | F : 5’-gtatttttgcaaggctaccagttacatttgacaa-3’ Tm=55,8°C  R : 5’-gattggcttcaggatgtccatgttggaa-3’ Tm=56,1°C | ***EcoNI*** |
| ***APOE***  **ε2 / ε3 / ε4**  ***(rs7412, rs429358)*** | F : 5’ –TAAGCTTGGCACGGCTGTCCAAGGA– 3’ Tm=56,8°C  R: 5’–ACAGAATTCGCCCCGGCCTGGTACAC– 3’ Tm=59,3°C | ***HhaI*** |
| ***PPARG^161C/T^***  ***(rs3876806)*** | F : 5’-CAAGACAACCTGCTACAAGC-3’ Tm=60°C  R : 5’-tccttgtagatctcctgcag-3’ Tm=60°C | ***PmlI*** |
| ***SELE^561A/C^ (rs5361)*** | F : 5’-AGTAATAGTCCTCCTCATCATG-3’ Tm=62°C  R : 5’–ACCATCTCAAGTGAAGAAAGAG–3’ Tm=62°C | ***PstI*** |
| ***ITGA2 (gpIa^807C/T, 837C/T, 873 G/A^*** *^*^****)***  ***(rs1126643, 1139484, 1062535)*** | F : 5’-CTCTCTAGATTGTCATGGTTGCATTGATCAATCAC– 3’ Tm=57,7°C  R : 5’ –GATTTAACTTTCCCGACTGCCTTC– 3’ Tm=52,7°C | ***Bgl II***  ***Asn I*** |
| ***ITGB3^L33P^ (gpIIIa)***  ***(rs5918)*** | F : 5’ –GTGCAGGAGGTAGAGAGTCGCCATAG- 3’ Tm = 58,1°C  R : 5’ –GTG-CAATCCTCTGGGGACTGACTT-G– 3’ Tm = 56,8°C | ***ScrfI*** |
